# Association Between Complete Blood Count and the Lipoxygenase Pathway in Hashimoto’s Thyroiditis

**DOI:** 10.3390/cells14241933

**Published:** 2025-12-05

**Authors:** Karolina Wrońska, Maciej Ziętek, Tomasz Machałowski, Małgorzata Szczuko

**Affiliations:** 1 Department of Bromatology and Nutritional Diagnostics, Pomeranian Medical University in Szczecin, 71-460 Szczecin, Poland; 74985@student.pum.edu.pl; 2 Department of General Pharmacology and Pharmacoeconomics, Pomeranian Medical University in Szczecin, 71-460 Szczecin, Poland; maciej.zietek@pum.edu.pl; 3 Department of Perinatology, Obstetrics and Gynecology, Pomeranian Medical University in Szczecin, 70-204 Szczecin, Poland; tomasz.machalowski@pum.edu.pl

**Keywords:** Hashimoto’s Thyroiditis, AA, LOX, HETE, LT, LX blood count, inflammation

## Abstract

**Highlights:**

**What are the main findings?**

- Pro-inflammatory arachidonic acid (AA) derivatives may play a vital role in the development of Hashimoto’s Thyroiditis (HT) and the production of anti-thyroid peroxidase antibodies (ATPO) and anti-thyroglobulin antibodies (ATG).
- Excessive activation of lipoxygenases (LOX) in HT may lead to increased pro-inflammatory hydroxyeico-satetraenoic acids (5S-HETE, 12S-HETE and 15S-HETE) and 5-oxo-eicosatetraenoic acid (5-oxo-ETE).

**What are the implications of the main findings?**

- It may be associated with the development of the most wide-spread endocrine cancer in the world, thyroid cancer.
- This study may contribute to developing new guidelines for diagnosing and treating HT and other autoimmune diseases.

**Abstract:**

Background: Hashimoto’s Thyroiditis (HT) is one of the most common autoimmune diseases worldwide, yet little is known about the role of lipid mediators in its pathogenesis. This study investigated whether there is a link between complete blood count (CBC) and arachidonic acid (AA) derivatives resulting from the activation of lipoxygenases (LOX) in 39 female patients with HT. Material and Methods: Blood samples were used as the research material. Liquid chromatography was employed to analyze the lipid mediators. Results: Neutropenia, lymphopenia and basopenia were observed in the women studied. An increase in mean corpuscular volume (MCV) and low haematocrit (HCT) and hemoglobin (HGB) levels were also noted. The highest amounts of hydroxyeicosatetraenoic acids (5S-HETE, 12S-HETE and 15S-HETE) and 5-oxo-eicosatetraenoic acid (5-oxo-ETE) were observed in the study group. The strongest positive correlations were observed between the acids and C-reactive protein (CRP), neutrophils (NEUT), and eosinophils (EOS). Furthermore, significant correlations between eicosanoids and anthropometric parameters were also presented. Conclusions: Eicosanoids may play a crucial role in the pathogenesis of HT, affecting complete blood count. Further research in this area could lead to the development of new diagnostic markers and therapeutic strategies, including those aimed at the anticancer treatment of this gland.

## 1. Introduction

### 1.1. Hashimoto’s Thyroiditis—Epidemiology, Etiology, and Pathogenesis

Hashimoto’s Thyroiditis (HT) also known as chronic lymphocytic thyroiditis is one of the most common autoimmune diseases in the world. It affects approximately 5% of the population, and the number of cases is constantly growing, especially in developed Western countries [1]. The etiology of the disease is complex. The disease is 7–10 times more common in women than in men, with the highest incidence occurring between the ages of 30 and 60 [2,3]. There are many genetic, environmental, and immunological factors that increase the risk of HT. Polymorphisms of genes contributing to the loss of immune tolerance have been discovered, such as: human leukocyte antigens (HLA-DR), cytotoxic T-cell antigen-4 (CTLA-4), cell differentiation antigen 25 (CD25), cell differentiation antigen 40 (CD40), non-receptor tyrosine phosphatase type 22 (PTPN22) and forkhead transcription protein P3 (FOXP3) [4]. Environmental determinants influencing the onset of Hashimoto’s Thyroiditis include smoking, infections, medications, toxins, alcohol, stress, and pregnancy [5]. Nutrition and gut microbiota are other important factors that modulate the immune response, affect nutrient absorption, and epigenetic mechanisms regulating the synthesis and metabolism of thyroid hormones [6].

**Figure 1:**
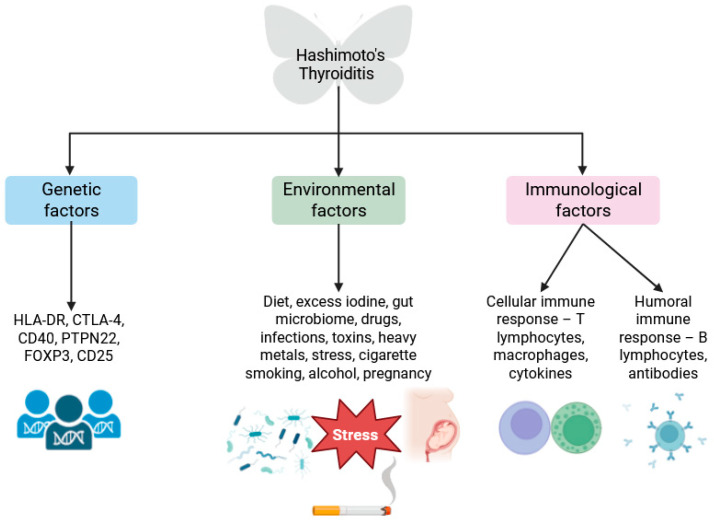
Pathogenesis of Hashimoto’s Thyroiditis. Created in BioRender. Wrońska K. (2025) https://app.biorender.com/illustrations/673bb726c1abf396d5aa0e79.

Chronic lymphocytic thyroiditis is associated with an abnormal immune response, excessive activation of the immune system, and the formation of autoreactive cells. CD4+ and CD8+ T lymphocytes, as well as CD19+ B lymphocytes, macrophages, and plasma cells infiltrate the thyroid gland. Helper T cells (Th)—Th1 and Th17 are particularly involved in the pathogenesis of the disease. Cytotoxic T cells (Tc) are also crucial in the course of HT, as they destroy the thyroid gland and exacerbate the course of the disease by producing cytotoxic components such as perforin, granzymes, and proteoglycans [7]. The deficiency and abnormal activity of regulatory T cells (Treg) in Hashimoto’s Thyroiditis, confirmed in studies, may be an important factor in the development of the disease by disrupting immune tolerance to the body’s own antigens [8]. B lymphocytes, on the other hand, are associated with the humoral response and are the main source of antibodies against thyroid peroxidase (ATPO) and thyroglobulin (ATG) [9]. An increase in pro-inflammatory interleukins (IL) such as IL-1β, IL-4, IL-6, IL-8, IL-12, IL-14, IL-17, IL-21, IL-22, as well as tumor necrosis factor-α (TNF-α) and interferon gamma (IFN-γ) was also observed in the thyrocytes of patients with autoimmune thyroid diseases (AITD) [10]. CD20+ T lymphocytes, which are capable of producing larger amounts of pro-inflammatory mediators such as IL-17-A and IFN-γ than CD20 T lymphocytes, may play a significant role in the inflammatory process in Hashimoto’s Thyroiditis [11]. Chronic inflammation and infiltration of the gland by pro-inflammatory cells and mediators leads to the destruction of the thyroid epithelium and thyrocytes, resulting in glandular hypofunction and insufficient hormone secretion [12]. A summary of the pathogenesis of Hashimoto’s Thyroiditis is presented in Figure 1.

### 1.2. Lipoxygenase-Derived Products

Lipids are a group of organic compounds with hydrophobic properties and low solubility in water [13]. They constitute 10–20% of the human body and are part of cell membranes [14]. They perform many important functions in the human body, such as energy storage and thermal insulation. They also participate in the regulation of cell structure and function, signaling pathways, and gene expression [15]. Any abnormalities in lipid metabolism and their excessive accumulation in tissues and organs lead to cell dysfunction and death [16].

Omega-3 acids with the first double bond located on the third carbon include: α-linolenic acid (ALA), eicosapentaenoic acid (EPA), docosahexaenoic acid (DHA), and docosapentaenoic acid (DPA). Omega-6 fatty acids with the first double bond on the sixth carbon include: linoleic acid (LA; 18:2n-6), gamma-linolenic acid (GLA; 18:3n-6), dihomo-gamma-linolenic acid (DGLA; 20:3n-6) and arachidonic acid (AA; 20:4n-6) [17,18]. Arachidonic acid is a ubiquitous component of phospholipids in cell membranes, affecting their fluidity. It is mainly obtained by humans from food, but is also synthesized as a result of LA metabolism [19]. In addition to its building function, this acid is the main precursor used for the synthesis of pro-inflammatory lipid mediators [20]. Furthermore, it plays a crucial role in the initiation and modification of the inflammatory response [21]. Linoleic acid also has pro-inflammatory effects [22]. EPA, DHA, and DPA, on the other hand, have anti-inflammatory and protective effects and are converted into specialized anti-inflammatory mediators (SPMs), which include resolvins, protectins, and maresins [23].

**Figure 2:**
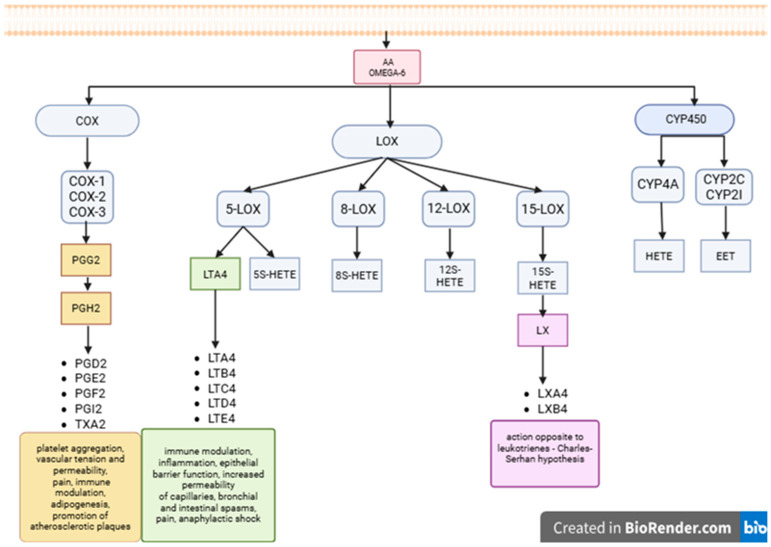
The arachidonic acid metabolism pathways. AA—arachidonic acid; COX—cyclooxygenase; CYP—cytochrome P450; EET—epoxyicosatrienoic acid; HETE—hydroxyeicosatetraenoic acid; LOX—lipoxygenase; LT (data marked in green)—leukotrienes; LX (data marked in purple)—lipoxins; PG (data marked in orange)—prostaglandins; PGI2—prostacyclin; TX—thromboxanes; Created in BioRender. Wrońska K and Szczuko M. (2025). https://app.biorender.com/illustrations/673ba0631322cf7327fa131e.

Eicosanoids are derivatives of polyunsaturated fatty acids, including arachidonic acid. These lipid signaling mediators are involved in physiological and pathological processes. They are responsible for cellular metabolism and apoptosis [24]. They also play a crucial role in modulating the immune system, controlling inflammatory processes through chemotaxis and leukocyte activation, fever, pain, blood vessel permeability and tension, as well as platelet aggregation [25]. Arachidonic acid is a precursor of dienes with high biological activity that modify the immune response [26]. The synthesis of lipid mediators is initiated by the activation of phospholipase A2 (PLA2), which responds to various stimuli, including inflammatory stimuli. With its help, arachidonic acid bound to the cell membrane is released from phospholipids into the cytoplasm [27]. It is then converted into potent inflammatory mediators such as prostaglandins (PG), leukotrienes (LT), thromboxanes (TX), and hydroxyeicosatetraenoic acids (HETEs) [28]. Lipid mediators can be formed not only from AA, but also from LA, and include hydroxyoctadecadienoic acids (HODE) [29]. Eicosanoids are formed as a result of the metabolism of arachidonic acid through three main pathways involving cyclooxygenase (COX), lipoxygenase (LOX), and cytochrome P450 (CYP) enzymes [30]. The first pathway- COX-1, COX-2, and COX-3—leads to the formation of prostanoids—prostaglandins H2, E2, D2 and F2 (PGH2, PGE2, PGD2, PGF2), prostacyclins (PGI2), and thromboxane A2 (TXA2)—as a result of the conversion of AA to prostaglandin G2 (PGG2) and then PGH2 [31]. In the second pathway—lipoxygenase pathway, which is currently used for therapeutic purposes, the following can be distinguished: 5-LOX, 8-LOX, 12-LOX, and 15-LOX, which initiate the production of leukotrienes A4, B4, C4 and E4 (LTA4, LTB4, LTC4, LTD4, LTE4), lipoxins A4 and B4 (LXA4, LXB4), and 5S-, 8S-, 12S-, 15S-hydroxyeicosatetraenoic acids (5S-HETE, 8S-HETE, 12S-HETE, 15S-HETE) [32]. Oxidation by 5-hydroxyecosanoyl dehydrogenase of 5S-HETE acid produced by 5-LOX leads to the production of 5-oxo-eicosatetraenoic acid (5-oxo-ETE) [33]. The third pathway of pro-inflammatory arachidonic acid derivative synthesis is associated with CYP-450 activation and leads to the formation of epoxy eicosatrienoic acids (EETs), such as 5,6-EET, 8,9-EET, 11-12-EET, and 14,15-EET. In contrast, the action of CYP-450 and ω-hydroxylase results in the production of HETE acids (19S- and 20S-HETE) [34]. The arachidonic acid metabolism pathways is summarized on Figure 2.

The aim of this study was to demonstrate the relationship between hematological parameters and pro-inflammatory arachidonic acid derivatives resulting from LOX pathway activation in women suffering from Hashimoto’s Thyroiditis, and to improve our understanding of the role of lipid mediators in the pathogenesis of autoimmune diseases.

## 2. Materials and Methods

### 2.1. Characteristics of the Study Group

The study group included 39 Caucasian women with Hashimoto’s Thyroiditis aged 37.395 ± 8.959 years, who were admitted to the Department of Endocrinology, Metabolic Diseases, and Internal Medicine at the Pomeranian Medical University in Szczecin. The diagnosis was made within the last three years based on thyroid ultrasound (USG) examination and elevated anti-thyroid antibodies concentrations in the blood. The inclusion criteria for the study were: female gender, reproductive age, and chronic lymphocytic thyroiditis diagnosed on the basis of ultrasound imaging and ATPO and ATG antibody blood concentrations. The exclusion criteria for participation in this study were as follows: surgical removal of the thyroid gland, Graves’ disease, metabolic diseases such as diabetes, hypertension, ischemic heart disease, use of medications such as immunosuppressants, nonsteroidal anti-inflammatory drugs, and medications affecting thyroid function other than levothyroxine. Blood samples were taken from the women for testing, and complete blood count (CBC), C-reactive protein (CRP), eicosanoid levels, and biochemical parameters were analyzed to assess thyroid function (ATPO, ATG, thyroid stimulating hormone (TSH), free triiodothyronine (FT3), and free thyroxine (FT4)) [35]. In addition, anthropometric measurements were taken during the screening visit. Levothyroxine was administered to 36 patients to normalize thyroid parameters. The study was conducted in accordance with the Declaration of Helsinki. The study received approval from the Bioethical Commission of the Pomeranian Medical Uni-versity in Szczecin (approval numbers KB-0012/145/17, date 6 November 2017).

### 2.2. Sample Collection

Fasting blood samples were collected from each patient into 10 mL polypropylene tubes containing ethylenediaminetetraacetic acid (EDTA). The obtained test material was then centrifuged using a refrigerated centrifuge for 10 min (3000 rpm). After centrifugation, the plasma was separated into 0.5 mL Eppendorf tubes and stored in a freezer at −80 °C until analysis. Electrochemiluminescence immunoassay (ECLIA) was used to measure thyroid parameters (TSH, FT3, FT4, ATPO, and ATG) in the patients’ blood serum using a Roche Cobas model 6000 module 601 device (Indianapolis, IN, USA) [12].

### 2.3. Eicosanoid Extraction

Arachidonic and linoleic acid derivatives were extracted from blood serum using RP-18 SPE columns for solid-phase extraction (Agilent Technologies, Cheadle, UK): 5(S),6(R)-Lipoxin A4 (LXA4 5S. 6R), 15(R)-Lipoxin A4 (LXA4 5S. 6R. 15R), 5S-HETE, 5-oxo-ETE, 12S-HETE, 15S-HETE, and Leukotriene B4 (LTB4). During the extraction of the tested lipid mediators, 0.5 mL of the obtained plasma was added to 1 mL of acetonitrile to precipitate the protein. 50 μL of internal standard (1 μg/mL) was also added. The mixture was then incubated for 15 min at −20 °C. In the next step, the samples were centrifuged for 10 min at 10,000 rpm using a refrigerated centrifuge (Eppendorf, Hamburg, Germany, Centrifuge 5804R). The material obtained in this way was transferred to new tubes, and then 4.5 mL of 1 mM HCl was added. Each sample was adjusted to pH = 3 by adding 30–50 μL of 1 M HCl. The columns were activated by successive rinses with 3 mL of 100% acetonitrile and 3 mL of 20% acetonitrile in water. The samples were then loaded and washed twice in water using 3 mL of 20% acetonitrile. In the process of eluting eicosanoids, 1.5 mL of a mixture of methanol and ethyl acetate (1/1 *v*/*v*) was added, dried under vacuum, and dissolved in 100 μL of 60% methanol in water with the addition of 0.1% acetic acid. Each sample obtained in the manner described above was immediately analyzed using a high-performance liquid chromatography (HPLC) device [36].

### 2.4. HPLC Operating Parameters

An Agilent Technologies 1260 liquid chromatograph was used for HPLC separation, consisting of a degasser, a reservoir pump, a column oven, and a diode array detector (DAD). Agilent ChemStation software (B.0404; Agilent Technologies, Cheadle, UK) was utilized. Separation was performed with a Thermo Scientific Hypersil BDS C18 column (100 × 4.6 mm, 2.4 μm; cat. no. 28102-154630). The column oven temperature was set to 20 °C. A gradient method was used for the analysis. The mobile phase consisted of a mixture of solvent A (methanol/water/acetic acid, 50/50/0.1, *v*/*v*/*v*) and B (methanol/water/acetic acid, 100/0/0.1, *v*/*v*/*v*). In the mobile phase, the buffer B content was 30% during the first 2 min of separation. This was followed by a linear increase to 80% over 33 min. 98% was obtained between 33.1 and 37.5 min, and 30% was recorded between 40.3 and 45 min. The flow rate was 1.0 mL/min and the sample injection volume was 60 µL. DAD monitored the peaks by adsorption, and the absorption spectra were analyzed to confirm the identification of the analytes. Quantitative determination was based on peak areas with internal standard calibration [36].

### 2.5. Statistical Analysis

Statistica 13 (Statsoft, Krakow, Poland) was utilized to execute statistical analyses.

The Shapiro–Wilk test was used to check the normality of the distribution. Most of the data analyzed did not have a normal distribution, so Spearman’s correlation test was performed. The result was considered statistically significant if *p* < 0.05.

## 3. Results

### 3.1. Analysis of the Study Group

The average age of the patients was 37.395 ± 8.959 years. Overweight (Body mass index (BMI): 25.0–29.9 kg/m^2^) was found in 14 women, obesity I degree (BMI: 30.0–34.9 kg/m^2^) in 6 women, and obesity II degree (BMI: 35.0–39.9 kg/m^2^) in one patient. The average BMI for the group of women studied was 25.739 ± 4.417 kg/m^2^. Abnormal anthropometric measurements were found, such as excessive body weight, fat tissue mass and body fat percentage. An analysis of biochemical parameters was also performed to evaluate thyroid function and the severity of Hashimoto’s Thyroiditis. Elevated levels of antibodies against ATPO and ATG were observed in 56.4% of patients. Elevated levels of antibodies against ATPO alone were observed in 35.9% of patients, and elevated levels of antibodies against ATG alone were observed in 7.7% of patients. The average ATPO level in the women studied was 228.581 ± 290.014 IU/mL, while for ATG antibodies it was 319.631 ± 546.504 IU/mL. Hypothyroidism (↑TSH and ↓FT4 and FT3) was detected in 1 patient. Hyperthyroidism was also detected in one of the women studied (↓TSH and ↑FT3). The average TSH level in patients was 3.041 ± 2.748 µIU/mL. The average FT3 level was 2.985 ± 0.565 pg/mL, while FT4 was 1.284 ± 0.196 ng/dL. Levothyroxine was taken by 92.3% of the subjects (in doses ranging from 25 to 150 µg), with only 3 women not undergoing therapy. Positive correlations were obtained between FT4 and percentage body fat (r = 0.272; *p* = 0.094), and fat tissue mass (r = 0.213; *p* = 0.192). In addition, a negative correlation was found between FT3 and soft lean body mass (r = −0.312; *p* = 0.05). Data on the characteristics and anthropometric measurements of the study group, the biochemical parameters analyzed, and the levothyroxine dose are presented in Table 1.

### 3.2. Analysis of Complete Blood Count and C-Reactive Protein in the Study Group

An analysis of the complete blood count of the patients examined and the CRP index was performed. The average CRP in the group was 1.449 ± 1.439 mg/L. Abnormalities in CBC were found. Neutropenia was detected in 14 of the women examined, lymphopenia in 8, and basopenia in 8. A decrease in red blood cell parameters such as hemoglobin and haematocrit was also observed. An increase in the mean red blood cell volume was noted in 7 patients. Significant correlations between thyroid parameters and complete blood count were observed. In the case of ATPO, negative correlations with basophils (r = −0.406; *p* = 0.013) and eosinophils (r = −0.272; *p* = 0.103) were noted. Moreover, a positive correlation between ATG and neutrophils (r = 0.224; *p* = 0.182) was noted. TSH levels correlated positively with monocyte levels (r = 0.296; *p* = 0.075), and platelets (r = 0.249; *p* = 0.137), and negatively with haematocrit (r = −0.472; *p* = 0.003), hemoglobin (r = −0.403; *p* = 0.013), and erythrocytes (r = −0.332; *p* = 0.045). Positive correlations were also found between FT4 and erythrocytes (r = 0.384; *p* = 0.019), haematocrit (r = 0.344; *p* = 0.037), and eosinophils (r = 0.268; *p* = 0.108). In addition, the relationship between the anthropometric parameters of the patients and their complete blood count was analyzed. Significant correlations were observed between fat tissue mass, % body fat content, BMI, body weight and the CRP index (respectively: r = 0.581 *p* = 0.000; r = 0.565; *p* = 0.000; r = 0.557; *p* = 0.000; r = 0.527; *p* = 0.001). In addition, positive correlations were observed between body weight and erythrocytes (r = 0.332; *p* = 0.044); BMI and neutrophils (r = 0.312; *p* = 0.061), and leukocytes (r = 0.305; *p* = 0.066); fat tissue mass and neutrophils (r = 0.351; *p* = 0.034), erythrocytes (r = 0.342; *p* = 0.038), and leukocytes (r = 0.322; *p* = 0.052). In turn, % body fat content correlated positively with neutrophils (r = 0.343; *p* = 0.038), and leukocytes (r = 0.315; *p* = 0.057). A summary of CBC and CRP analysis is presented in Table 2.

### 3.3. Characteristics of Arachidonic Acids Derivatives in HT Patients

**Figure 3:**
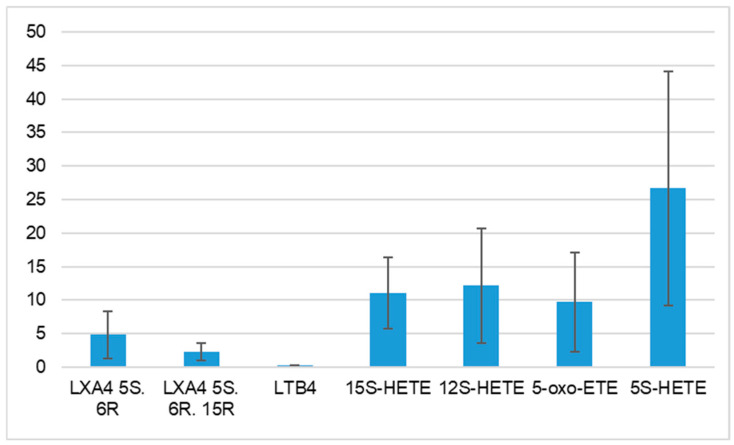
Products of the lipoxygenase pathway in Hashimoto’s Thyroiditis [mean value ± standard deviation; μg/mL]. LX—lipoxins; LT—leukotrienes; HETE—hydroxyeicosatetraenoic acid; 5–oxo–ETE—5–oxo–eicosatetraen-oic acid.

After measuring pro-inflammatory derivatives of AA in the blood of women suffering from Hashimoto’s Thyroiditis, the highest average values of the following pro-inflammatory lipid mediators were found: 5S-HETE, 12S-HETE, 15S-HETE, and 5-oxo-ETE. In turn, the lowest amounts were recorded for: LTB4, LXA4 5S. 6R. 15R and LXA4 5S. 6R. A summary of the results obtained for AA derivatives associated with LOX activation is presented in Figure 3.

Importantly, positive correlations were observed between the anthropometric parameters of patients and the arachidonic acid derivatives. Patients who were overweight or obese had significantly higher levels of 5S-HETE, LXA4 5S. 6R. 15R, LTB4, 15S-HETE, and 5-oxo-ETE. The most significant positive correlations were obtained for:

- LTB4 and fat tissue mass (r = 0.352; *p* = 0.035)
- LXA4 5S. 6R. 15R and body weight (r = 0.488; *p* = 0.002)
- LXA4 5S. 6R. 15R and BMI (r = 0.484; *p* = 0.003)
- LXA4 5S. 6R. 15R and fat tissue mass (r = 0.545; *p* = 0.000)
- LXA4 5S. 6R. 15R and % body fat content (r = 0.525; *p* = 0.001)
- 5S-HETE and body weight (r = 0.399; *p* = 0.016)
- 5S-HETE and BMI (r = 0.346; *p* = 0.039)
- 5S-HETE and fat tissue mass (r = 0.415; *p* = 0.012)
- 5S-HETE and % body fat content (r = 0.369; *p* = 0.027)
- 5S-HETE and soft lean mass (r = 0.337; *p* = 0.044)
- 15S-HETE and fat tissue mass (r = 0.307; *p* = 0.068)
- 5-oxo-ETE and fat tissue mass (r = 0.297; *p* = 0.078)

Additionally, negative correlations were also observed between lipid mediators and anti-thyroid antibodies: LTB4 and ATPO (r = −0.296; *p* = 0.068), and 12S-HETE and ATPO (r = −0.265; *p* = 0.103). On the other hand, a positive correlation was observed between LXA4 5S. 6R. 15R and FT4 (r = 0.245; *p* = 0.132).

### 3.4. Correlations Between Complete Blood Count and C-Reactive Protein with Arachidonic Acids Derivatives in HT Patients

After performing Spearman’s correlation analysis, a relationship was observed between the acids and white blood cell, red blood cell, and platelet parameters.

Positive correlations were obtained for:

- neutrophils and LXA4 5S. 6R. 15R (r = 0.339; *p* = 0.039)
- eosinophils and LXA4 5S. 6R. 15R (r = 0.304; *p* = 0.068)
- erythrocytes and 15S-HETE (r = 0.299; *p* = 0.071)
- neutrophils and LTB4 (r = 0.287; *p* = 0.085)
- neutrophils and 5-oxo-ETE (r = 0.277; *p* = 0.097)
- leukocytes and LXA4 5S. 6R. 15R (r = 0.275; *p* = 0.099)
- monocytes and LXA4 5S. 6R. 15R (r = 0.267; *p* = 0.111)
- eosinophils and 15S-HETE (r = 0.257; *p* = 0.124)
- eosinophils and LTB4 (r = 0.244; *p* = 0.145)
- leukocytes and LTB4 (r = 0.237; *p* = 0.158)
- leukocytes and 5-oxo-ETE (r = 0.231; *p* = 0.169)
- eosinophils and 5-oxo-ETE (r = 0.216; *p* = 0.201)
- erythrocytes and LXA4 5S. 6R. 15R (r = 0.215; *p* = 0.202)

In addition, negative correlations were found between CBC and AA derivatives, although these correlations were fewer and weaker compared to the above correlations. A correlation was observed between:

- platelets and 15S-HETE (r = −0.293; *p* = 0.079)
- erythrocytes and LXA4 5S. 6R (r = −0.236; *p* = 0.161)
- platelets and LXA4 5S. 6R (r = −0.222; *p* = 0.187)
- mean red blood cell volume and LXA4 5S. 6R. 15R (r = −0.219; *p* = 0.191)
- mean red blood cell volume and 15S-HETE (r = −0.212; *p* = 0.208)
- lymphocytes and 12S-HETE (r = −0.194; *p* = 0.249)
- mean red blood cell volume and 12S-HETE (r = −0.163; *p* = 0.334)

**Figure 4:**
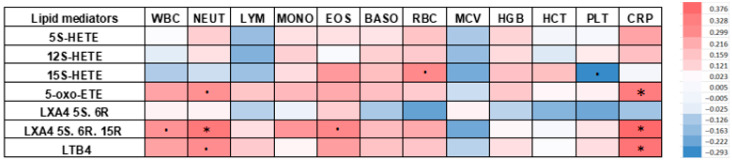
Correlation of lipid mediators with complete blood count and C-reactive protein in women with Hashimoto’s Thyroiditis (Positive correlations are shown in red and negative correlations in blue. The darker the color, the greater the statistical significance.). HETE—hydroxyeicosatetraenoic acid; 5–oxo–ETE—5–oxo–eicosatetraenoic acid; LX—lipoxins; LT—leukotrienes; WBC—leukocytes; NEUT—neutrophils; LYM—lymphocytes; MONO—monocytes; EOS—eosinophils; BASO—basophils; RBC—erythrocytes; MCV—mean corpuscular volume; HGB—hemoglobin; HCT—haematocrit; PLT—platelets; CRP—C-reactive protein; *—statistically significant result (*p* < 0.05); ●—statistically significant result at the trend level (0.05 < *p* < 0.1).

**Figure 5:**
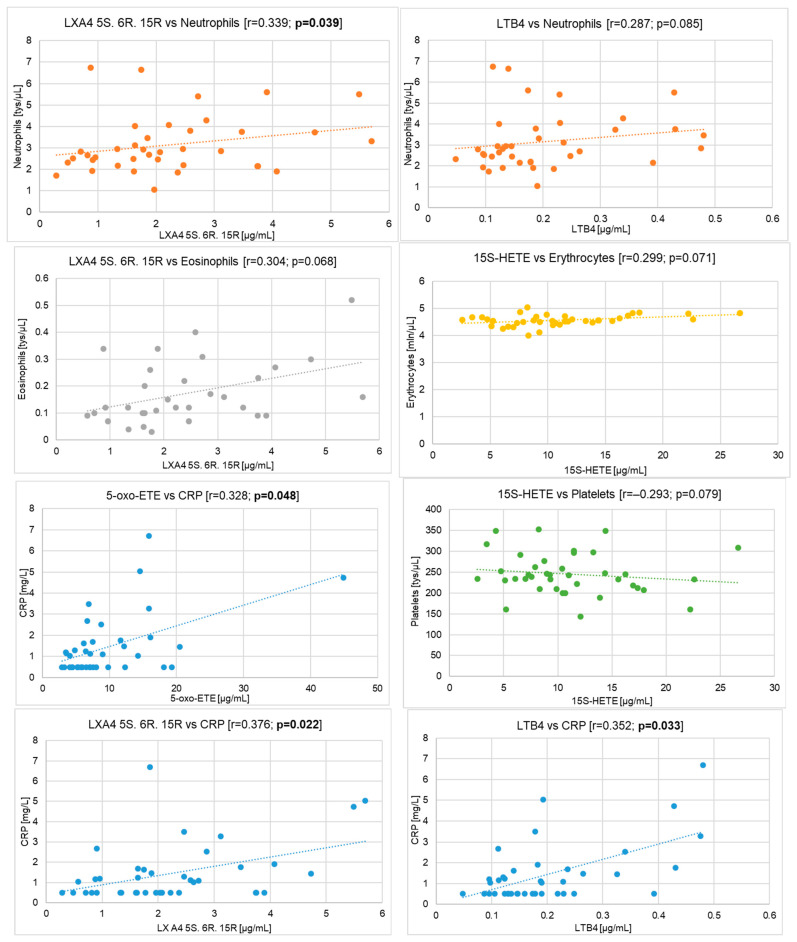
The most significant correlations between eicosanoids and complete blood count and C-reactive protein in women with Hashimoto’s Thyroiditis. Each blood morphology parameter and CRP are shown in a different colour on the graph (orange—neutrophils; grey—eosinophils; yellow —erythrocytes; blue—CRP; green—platelets).

After measuring lipid mediators and CRP, their mutual relationship was also examined. The significant positive correlations were obtained between CRP and LXA4 5S. 6R. 15R (r = 0.376; *p* = 0.022), LTB4 (r = 0.352; *p* = 0.033), 5-oxo-ETE (r = 0.328; *p* = 0.048), and 5S-HETE (r = 0.231; *p* = 0.169). The correlations obtained between AA acid derivatives and CBC and CRP in patients suffering from Hashimoto’s Thyroiditis appear to be significant, especially in the case of 5S-HETE, and 5-oxo-ETE, due to the highest amounts of these acids in the blood of the tested women. The correlations described above are presented in Table 3 and Figure 4 in the form of a heat map. The most statistically significant correlations between eicosanoids and CBC and CRP were also shown in scatter plots (Figure 5).

**Figure 6:**
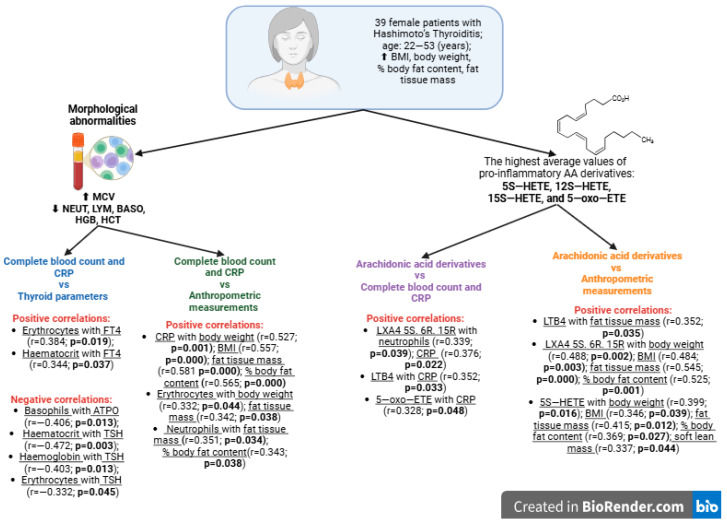
Summary of the study results. BMI—body mass index; MCV—mean corpuscular volume; NEUT—neutrophils; LYM—lymphocytes; BASO—basophils; HGB—hemoglobin; HCT—haematocrit; CRP—C-reactive protein; FT4—free thyroxine; r—Spearman correlation; *p*—*p*-value; ATPO—anti-thyroid peroxidase antibodies; TSH—thyroid-stimulating hormone; HETE—hydroxyeicosatetraenoic acid; 5–oxo–ETE—5–oxo–eicosatetraenoic acid; LX—lipoxins; LT—leukotrienes; ⬆—increase; ⬇—decrease; Bold font indicates statistically significant correlation (*p* < 0.05); Created in BioRender. Wrońska K (2025); https://app.biorender.com/illustrations/65f084c6e21815e3714c195e.

It is also worth noting the correlations between neutrophil to lymphocyte ratio (NLR) and platelet to lymphocyte ratio (PLR) and AA derivatives. In the study group of patients, positive correlations were observed between NLR and 5S-HETE (r = 0.231; *p* = 0.168) and 12S-HETE (r = 0.225; *p* = 0.182). In the case of PLR, there was a positive correlation with 12S-HETE (r = 0.195; *p* = 0.247) and a negative correlation with LXA4 5S. 6R (r = −0.324; *p* = 0.05). A comprehensive summary of the study results is presented in Figure 6.

## 4. Discussion

### 4.1. Pro–Inflammatory Lipid Mediators in the Course of Autoimmune Diseases

This study is one of the first to investigate the association between complete blood count and arachidonic acid derivatives in women with Hashimoto’s Thyroiditis. Randomized studies and a meta–analysis conducted in 2024 by Wang et al. confirmed the involvement of lipid metabolites in the pathogenesis of autoimmune diseases such as systemic lupus erythematosus, rheumatoid arthritis, inflammatory bowel disease, type I diabetes, and celiac disease [37]. Lipid mediators, including AA derivatives, can affect immune cells by influencing peroxisome proliferator–activated receptors (PPAR) and hepatic receptor X [38]. The incidence of Hashimoto’s Thyroiditis and thyroid cancer has been linked, among others, by Czapiewska et al. In their study, they showed that lipid concentrations are higher in the tissues of people suffering from both papillary thyroid cancer and Hashimoto’s Thyroiditis compared to people with papillary thyroid cancer alone [39]. In addition, in patients with HT increased FA 2 desaturase activity and changes in 15–LOX expression were observed [39]. In patients with papillary thyroid cancer, increased expression of 5–LOX and 12–LOX in glandular tissue was demonstrated, as well as their products: 5S–HETE and 12S–HETE. 12–LOX products play a vital role in the development of cancer and metastases by influencing cell proliferation and adhesion. It has also been shown that 12–LOX polymorphism (AG variant) is associated with an increased incidence of thyroid cancer, which should be taken into account in the diagnosis of patients with HT [40]. In this study, patients suffering from Hashimoto’s Thyroiditis showed increased synthesis of hydroxyeicosatetraenoic acids, in particular 5S–HETE, 12S–HETE, 15S–HETE, and 5–oxo–ETE. The increase in these mediators in the blood is associated with increased activity of 5–LOX, 12–LOX, and 15–LOX in patients with chronic lymphocytic thyroiditis and may indicate the involvement of the above–mentioned pathways in the pathogenesis of the disease. The activity of 12–LOX and 15–LOX was also increased in the course of other autoimmune diseases [41]. Scientific studies have shown the involvement of hydroxyeicosatetraenoic acids in the development of multiple sclerosis. An increase in 5S–HETE, 11S–HETE, 12S–HETE, and 15S–HETE was observed in patients, as well as a correlation between their levels and disease progression [41]. The activity of 12–LOX has been linked to immune cell migration and tissue damage, which is characteristic of autoimmune diseases [42]. This study also found an increase in 12S–HETE in women with Hashimoto’s Thyroiditis, confirming earlier reports suggesting increased levels of this eicosanoid in subclinical hypothyroidism. Other authors have observed a significant increase in 12S–HETE, especially in cases of TSH > 10.0 mIU/L [43]. Animal studies have also confirmed that 12/15–LOX is an inflammatory mediator that promotes the production of reactive oxygen species as a result of arachidonic acid metabolism [44]. The 5–LOX pathway may also be important in the pathogenesis of inflammatory diseases, as it is the main enzyme involved in the formation of pro–inflammatory AA derivatives such as leukotrienes, 5S–HETE, and 5–oxo–ETE [45]. Although the study found no significant correlation between the levels of pro–inflammatory arachidonic acid derivatives and FT3 and FT4, previous publications have shown a correlation. 20S–HETE, a metabolite produced by CYP450 activity, was positively correlated with thyroid hormones [46]. Our research indicates the involvement of pro-inflammatory derivatives in the pathogenesis of Hashimoto’s Thyroiditis, but further studies are needed to thoroughly analyze the correlation between eicosanoids and autoimmune diseases.

### 4.2. The Association Between Hashimoto’s Thyroiditis and Complete Blood Count

The study also presented the correlation between endocrine disorders associated with Hashimoto’s Thyroiditis and complete blood count of patients. The study group showed morphological abnormalities characteristic of HT. The most common abnormalities in the women studied were neutropenia (35.9%), lymphopenia (20.5%), and basopenia (20.5%). Abnormalities in red blood cell parameters, such as decreased hematocrit and hemoglobin, were also noted. The results obtained confirm earlier scientific reports. Ahmed and Mohammed showed that abnormal thyroid function has a negative effect on all blood morphometric parameters, with the weakest correlations observed in platelets [47]. A significant correlation was also found between abnormal thyroid function and red blood cell distribution width (RDW) (*p* = 0.002). An increase in this parameter often occurs in iron deficiency anemia, but also in vitamin B12 and folic acid deficiency, which are common in people with Hashimoto’s Thyroiditis and worsen the patient’s clinical condition [47]. In a study involving 144 patients with chronic lymphocytic thyroiditis, statistically significant differences in lymphocyte and neutrophil levels were observed in patients with HT compared to healthy individuals [48]. In a group of 84 women with HT and normal thyroid function, a decrease in lymphocyte count (*p* < 0.001) and lymphocyte percentage (*p* < 0.001) was observed, as well as an increase in neutrophil percentage (*p* < 0.001) compared to the control group. In contrast, in 60 women with HT and abnormal thyroid function, a decrease in lymphocyte count (*p* = 0.010) and percentage of lymphocytes (*p* < 0.001) and an increase in neutrophil count (*p* = 0.032) and percentage of neutrophils (*p* = 0.010) were observed. No correlation was observed between FT4 and TSH and the values of the parameters studied [48]. Contrary to the above results, in this study significant negative correlations were found between ATPO and basophils (r = −0.406; *p* = 0.013). Furthermore, TSH levels correlated negatively with haematocrit (r = −0.472; *p* = 0.003), hemoglobin (r = −0.403; *p* = 0.013), and erythrocytes (r = −0.332; *p* = 0.045). Positive correlations were also noted between FT4 and erythrocytes (r = 0.384; *p* = 0.019) and haematocrit (r = 0.344; *p* = 0.037). In a study by Tomczyńska et al., excessive platelet activity was observed in the vascular system of individuals with Hashimoto’s Thyroiditis and Graves’ disease [49]. The authors also demonstrated increased expression of the active form of the GPIIb/IIIa integrin receptor, which is associated with platelet aggregation. In addition, platelet aggregation was enhanced in various signaling pathways, including those dependent on adenosine diphosphate (ADP), collagen, and, importantly, arachidonic acid. An increase in aggregation as a result of AA was observed, and it was 20% higher in patients with HT (*p* < 0.005) compared to the group of people without autoimmune diseases [49]. In our study, we found a positive correlation between TSH and platelets (r = 0.249; *p* = 0.137). Although this correlation was not statistically significant, it may indicate an association between TSH and platelet count and activity, which may increase the risk of cardiovascular disease in patients with Hashimoto’s Thyroiditis. According to the results of our study, Önalan and Dönder have also found a decrease in haematocrit in patients with Hashimoto’s Thyroiditis compared to healthy individuals (*p* = 0.002) [50]. Neutropenia is another disorder in complete blood count, which occurs much more frequently in the course of autoimmune thyroid diseases, especially in individuals suffering from Hashimoto’s Thyroiditis (23.4% of patients) [51]. The results obtained are consistent with this study, in which neutropenia was observed in an even larger number of people—35.9%, which indicates the need to monitor the level of these cells in patients with Hashimoto’s Thyroiditis. Studies involving patients with chronic lymphocytic thyroiditis also reported an increase in CRP levels compared to a control group of healthy individuals (*p* = 0.064) [52]. In another scientific report on patients with hypothyroidism and HT, the authors also showed significantly higher CRP levels compared to individuals without HT (*p* < 0.001). The average CRP in this group of patients was 9.48 mg/dL [50]. Contrary to the above results, in this study, the mean CRP level in the patient group was not elevated, but the study was limited by the small number of participants. Moreover it is worth mentioning that significant correlations were observed between CRP and anthropometric parameters such as fat tissue mass (r = 0.581 *p* = 0.000), % body fat content (r = 0.565; *p* = 0.000), BMI (r = 0.557; *p* = 0.000) and body weight (r = 0.527; *p* = 0.001). The topic of the interrelationship between blood morphological abnormalities and Hashimoto’s Thyroiditis appears promising, and further research should be pursued to develop new diagnostic and therapeutic targets.

### 4.3. The Association Between Products of LOX and Complete Blood Count and CRP in Hashimoto’s Thyroiditis

There is a lack of data in the literature on the relationship between complete blood count and pro-inflammatory AA derivatives. Our study did not find many significant correlations between the eicosanoids and CBC, but the results were influenced by a small group of patients. After analyzing lipid mediators and complete blood count, the strongest positive correlations were found between neutrophils and LXA4 5S. 6R. 15R (r = 0.339; *p* = 0.039). We have not found confirmation in the literature of a positive correlation between LXA4 5S. 6R. 15R levels and neutrophil counts in patients with HT, so our report is the first to indicate the presence of such a relationship. Lipoxins are primarily involved in alleviating acute and chronic inflammation, reducing neutrophil activity and facilitating their elimination from the site of inflammation, and leading to a decrease in pro-inflammatory mediators from macrophages [53]. Lipoxins promote the migration of neutrophils to the site of inflammation by increasing their cytosolic calcium levels and help remove these cells from the site of inflammation, thereby alleviating inflammation. Balance in the life of neutrophils is vital because it has been shown that delayed neutrophil apoptosis can lead to inflammatory diseases and cancer [54]. Importantly, our study, in line with previous research findings, indicates a reduction in LXA4 levels in autoimmune diseases such as multiple sclerosis [55]. The positive correlation obtained between neutrophils and LXA4 5S. 6R. 15R, which exhibits anti-inflammatory activity, opens up new research perspectives and indicates the need for further studies to verify and expand existing knowledge on the pathogenesis of HT. In addition to lipoxin, positive correlations were observed between neutrophils and LTB4 (r = 0.287; *p* = 0.085) and 5-oxo-ETE (r = 0.277; *p* = 0.097). In earlier scientific reports showing also that 5–oxo–ETE has significant chemotactic activity on eosinophils, and after comparing all lipid mediators, 5–oxo–ETE was found to have the strongest effect [56]. Eosinophils can also convert arachidonic acid to 5–oxo–ETE. This pro–inflammatory eicosanoid can be released over a long period of time by inflammatory cells under conditions of oxidative stress, which promotes apoptosis. In addition, it is also a chemoattractant for other cells, such as neutrophils, monocytes, and basophils, and enhances the proliferation of cancer cells [56]. Furthermore, 5–oxo–ETE is responsible for stimulating basophil migration, leading to responses similar to those resulting from the action of chemokine CCL5 (RANTES), IL–8, or platelet–activating factor (PAF), and also induces a change in the shape of basophils. However, its ability to degranulate these cells has not been demonstrated [57]. When analyzing eosinophil levels, the strongest positive correlation was also found, as in the case of neutrophils, with LXA4 5S. 6R. 15R (r = 0.304; *p* = 0.068). As confirmed in this study, there are positive correlations between 5–oxo–ETE and neutrophils (r = 0.277; *p* = 0.097), eosinophils (r = 0.216; *p* = 0.201), monocytes (r = 0.175; *p* = 0.299), and basophils (r = 0.160; *p* = 0.343). However, none of the correlations mentioned were statistically significant. Additionally, a positive correlation between 15S-HETE and erythrocytes (r = 0.299; *p* = 0.071) was presented. In other scientific publications, authors have suggested that 15S-HETE may be considered a factor stimulating erythrocyte adhesion to endothelial cells, which in people with Hashimoto’s Thyroiditis may be significant in increasing the risk of cardiovascular disease, and therefore this relationship should be taken into account in the treatment of HT [58]. On the other hand negative effect of arachidonic acid on red blood cell parameters was also observed. High levels of AA led to oxidative stress and acute erythrocyte damage, resulting in a decrease not only in RBC levels but also in hemoglobin [59]. However, the study did not show strong negative correlations between RBC and HGB and the lipid mediators studied. A negative correlation was obtained only between RBC and LXA4 5S. 6R (r = −0.236; *p* = 0.161). Scientific reports have also confirmed the effect of AA on monocytes [60]. Damaged endothelium is associated with an increase in AA, which leads to increased monocyte activity. In addition, it has been shown that the presence of these cells in an environment exposed to high concentrations of arachidonic acid causes them to adopt a foamy phenotype, resulting in the accumulation of cytoplasmic lipid cells. Importantly, such cell transformation can significantly increase the risk of cardiovascular complications, therefore it is worth considering the relationship between monocytes and AA derivatives in the course of Hashimoto’s Thyroiditis [60]. We did not confirm significant correlation between arachidonic acid derivatives and monocytes in HT. In this study, the strongest negative correlation was observed between 15S-HETE and platelets (r = −0.293; *p* = 0.079). The literature has pointed to the role of 15-oxylipins, including 15S-HETE, in inhibiting platelet aggregation, suggesting that this is related to their effect on the common signaling process following receptor activation in the platelet aggregation pathway. However, it should be emphasized that the expression of 15–LOX–1 in platelets is low [61]. There is a lack of in–depth analyses confirming negative correlations between 15S-HETE and platelets in autoimmune diseases. A negative correlation was also observed between platelets and LXA4 5S. 6R (r = −0.222; *p* = 0.187). During therapy of patients with BML–11, which is an analog of LXA4, a decrease in platelet activity was demonstrated, which led to a reduction in the risk of thrombosis [62]. This study found correlations not only between arachidonic acid derivatives and CBC, but also CRP. The most significant correlations were presented between CRP and LXA4 5S. 6R. 15R (r = 0.376; *p* = 0.022), LTB4 (r = 0.352; *p* = 0.033), 5–oxo–ETE (r = 0.328; *p* = 0.048), and 5S–HETE (r = 0.231; *p* = 0.169). The results of the study confirm the finding described in the scientific literature. A study published in 2025 confirms a positive correlation between LTB4 and CRP in patients suffering from Hashimoto’s disease (before the gluten–free diet–r = 0.396; after the introduction of the gluten–free diet–r = 0.642) [63]. It is not possible to compare other correlations between eicosanoids and CRP due to the lack of available results from other authors. It can be assumed that the increase in pro–inflammatory lipid mediators such as 5–oxo–ETE, 5S–HETE and LTB4 in Hashimoto’s Thyroiditis and their positive correlation with the CRP is a vital mechanism associated with the modulation of the chronic inflammatory process. The number of articles describing the relationship between lipid mediators and CBC and CRP in autoimmune diseases is very limited. Our study is one of the first in the world and may be an important introduction to further research. The assessment of hematological parameters and pro–inflammatory arachidonic acid derivatives may be a vital supplement to the diagnosis of chronic lymphocytic thyroiditis and other inflammatory diseases. This study provides a new entry point for understanding the HT inflammatory regulatory network and the impact of LOX pathway products on complete blood count in females with HT. Although significant correlations were observed, the study has certain limitations that must be acknowledged. The main limitation of the study is the small number of females with HT. Therefore, the conducted observations should be expanded in the future to a larger study group. Additionally, there are other significant limitations that may affect the study’s results, such as the absence of a control group or the number of patients on levothyroxine therapy. Further analyses of the correlations between complete blood count, CRP and pro-inflammatory arachidonic acid derivatives produced by lipoxygenase pathway activation in patients with Hashimoto’s Thyroiditis are crucial and needed to understand the complex mechanisms leading to the development and exacerbation of HT.

## 5. Conclusions

The study’s findings indicate the presence of abnormalities in complete blood count during the process of inflammation leading to the destruction of thyrocytes. Patients with HT exhibit abnormalities such as neutropenia, lymphopenia, and basopenia. Pro–inflammatory arachidonic acid derivatives may play a vital role in the development of Hashimoto’s Thyroiditis and the production of ATG and ATPO antibodies. Excessive activation of 5–LOX, 12–LOX, and 15–LOX in HT may lead to increased pro–inflammatory eicosanoids such as 5S–HETE, 12S–HETE, 15S–HETE, and 5–oxo–ETE, which may contribute to thyroid cancer development. The levels of lipid mediators correlate with C–reactive protein, neutrophils, eosinophils, leukocytes, erythrocytes, platelets and mean red blood cell volume. Furthermore, this study confirmed the existence of statistically significant correlations between complete blood count and thyroid parameters and anthropometric parameters, as well as between eicosanoids and anthropometric parameters. These findings may contribute to a deeper understanding of the pathogenesis of Hashimoto’s Thyroiditis and other autoimmune diseases.

## Figures and Tables

**Table 1:** The characteristics of the study group—anthropometric measurements and biochemical parameters (n = 39).

Parameters	M ± SD [Min–Max]
Age [years]	37.395 ± 8.959 [22–53]
Height [cm]	166.615 ± 5.628 [150–178]
Body weight [kg]	71.521 ± 13.117 [51.6–110.2]
BMI [kg/m^2^]	25.739 ± 4.417 [18.28–37.25]
Fat tissue mass [g]	26.391.41 ± 9421.325 [1.954–54.316]
Body fat content [%]	35.888 ± 6.972 [20.12–49.30]
Soft lean mass [g]	42.686.051 ± 4749.222 [34.546–55.415]
ATPO [0–34 IU/mL]	228.581 ± 290.014 [9.34–1767]
ATG [0–115 IU/mL]	319.631 ± 546.504 [16.79–3423]
TSH [0.270–4.200 µIU/mL]	3.041 ± 2.748 [0.01–13.92]
FT3 [2.00–4.40 pg/mL]	2.985 ± 0.565 [1.78–4.87]
FT4 [0.93–1.70 ng/dL]	1.284 ± 0.196 [0.8–1.72]
Levothyroxine dose [µg]	68.792 ± 33.938 [25–150]

M—mean value; SD—standard deviation; Min—minimum; Max—maximum BMI—body mass index; ATPO—anti-thyroid peroxidase antibodies; ATG—anti-thyroglobulin antibodies; TSH—thyroid-stimulating hormone; FT3—free triiodothyronine; FT4—free thyroxine.

**Table 2:** Complete blood count and CRP analysis.

Parameters [Reference Range]	M ± SD [Min–Max]
CRP [0.0–5.0 mg/L]	1.449 ± 1.439 [<1–6.7]
WBC [3.8–10.00 tys/µL]	5.87 ± 1.691 [2.86–9.89]
NEUT [2.5–5.4 tys/µL]	3.153 ± 1.337 [1.05–6.74]
LYM [1.5–3.5 tys/µL]	2.007 ± 0.434 [1.02–2.83]
MONO [0.2–1.00 tys/µL]	0.528 ± 0.14 [0.27–0.87]
EOS [0.04–0.40 tys/µL]	0.173 ± 0.112 [0.03–0.52]
BASO [0.02– 0.10 tys/µL]	0.028 ± 0.015 [0.01–0.07]
RBC [3.7–5.10 mln/µL]	4.558 ± 0.207 [3.99–5.03]
HGB [12.0–16.0 g/dL]	13.346 ± 0.897 [10.3–15.1]
HCT [37.0–47.0%]	38.987 ± 2.264 [32.7–44]
MCV [80.0–90.0 fL]	85.564 ± 4.208 [71.9–92.7]
PLT [150–450 tys/µL]	245.769 ± 49.794 [143–353]

M—mean value; SD—standard deviation; Min—minimum; Max—maximum; CRP—C-reactive protein; WBC—leukocytes; NEUT—neutrophils; LYM—lymphocytes; MONO—monocytes; EOS—eosinophils; BASO—basophils; RBC—erythrocytes; HGB—hemoglobin; HCT—haematocrit; MCV—mean corpuscular volume; PLT—platelets.

**Table 3:** Correlation between lipid mediators and complete blood count and C-reactive protein.

	5S–HETE	12S–HETE	15S–HETE	5–oxo–ETE	LXA4 5S. 6R	LXA4 5S. 6R. 15R	LTB4
	[μg/mL]	[μg/mL]	[μg/mL]	[μg/mL]	[μg/mL]	[μg/mL]	[μg/mL]
WBC [tys/µL]	r = 0.005	r = −0.040	r = −0.126	r = 0.231	r = 0.026	r = 0.275	r = 0.237
	*p* = 0.978	*p* = 0.813	*p* = 0.457	*p* = 0.169	*p* = 0.879	*p* = 0.099	*p* = 0.158
NEUT [tys/µL]	r = 0.121	r = 0.076	r = −0.078	r = 0.277	r = 0.028	**r = 0.339**	r = 0.287
	*p* = 0.475	*p* = 0.655	*p* = 0.648	*p* = 0.097	*p* = 0.870	***p* = 0.039**	*p* = 0.085
LYM [tys/µL]	r = −0.162	r = −0.194	r = −0.154	r = 0.143	r = −0.111	r = 0.079	r = 0.132
	*p* = 0.338	*p* = 0.249	*p* = 0.362	*p* = 0.397	*p* = 0.513	*p* = 0.642	*p* = 0.435
MONO [tys/µL]	r = 0.074	r = 0.116	r = 0.085	r = 0.175	r = −0.025	r = 0.267	r = 0.023
	*p* = 0.662	*p* = 0.494	*p* = 0.616	*p* = 0.299	*p* = 0.884	*p* = 0.111	*p* = 0.895
EOS [tys/µL]	r = 0.074	r = −0.005	r = 0.257	r = 0.216	r = 0.121	r = 0.304	r = 0.244
	*p* = 0.665	*p* = 0.978	*p* = 0.124	*p* = 0.201	*p* = 0.475	*p* = 0.068	*p* = 0.145
BASO [tys/µL]	r = 0.066	r = 0.117	r = 0.163	r = 0.160	r = −0.133	r = 0.167	r = 0.150
	*p* = 0.697	*p* = 0.492	*p* = 0.335	*p* = 0.343	*p* = 0.432	*p* = 0.323	*p* = 0.376
RBC [mln/µL]	r = 0.148	r = 0.131	r = 0.299	r = 0.128	r = −0.236	r = 0.215	r = 0.145
	*p* = 0.381	*p* = 0.440	*p* = 0.071	*p* = 0.449	*p* = 0.161	*p* = 0.202	*p* = 0.393
MCV [fL]	r = −0.132	r = −0.163	r = −0.212	r = −0.080	r = 0.029	r = −0.219	r = −0.109
	*p* = 0.437	*p* = 0.334	*p* = 0.208	*p* = 0.636	*p* = 0.864	*p* = 0.191	*p* = 0.521
HGB [g/dL]	r = 0.106	r = 0.087	r = 0.154	r = 0.134	r = −0.105	r = 0.014	r = 0.079
	*p* = 0.533	*p* = 0.607	*p* = 0.362	*p* = 0.429	*p* = 0.537	*p* = 0.933	*p* = 0.644
HCT [%]	r = −0.013	r = −0.049	r = 0.158	r = 0.016	r = −0.189	r = −0.009	r = 0.004
	*p* = 0.941	*p* = 0.776	*p* = 0.351	*p* = 0.925	*p* = 0.262	*p* = 0.956	*p* = 0.981
PLT [tys/µL]	r = −0.010	r = 0.051	r = −0.293	r = 0.031	r = −0.222	r = 0.066	r = 0.084
	*p* = 0.951	*p* = 0.762	*p* = 0.079	*p* = 0.855	*p* = 0.187	*p* = 0.698	*p* = 0.622
CRP [mg/L]	r = 0.231	r = 0.159	r = −0.014	**r = 0.328**	r = −0.148	**r = 0.376**	**r = 0.352**
	*p* = 0.169	*p* = 0.346	*p* = 0.935	***p* = 0.048**	*p* = 0.382	***p* = 0.022**	***p* = 0.033**

r—Spearman correlation; *p*—*p*-value; HETE—hydroxyeicosatetraenoic acid; 5–oxo–ETE—5–oxo–eicosatetraenoic acid; LX—lipoxins; LT—leukotrienes; WBC—leukocytes; NEUT—neutrophils; LYM—lymphocytes; MONO—monocytes; EOS—eosinophils; BASO—basophils; RBC—erythrocytes; MCV—mean corpuscular volume; HGB—hemoglobin; HCT—haematocrit; PLT—platelets; CRP—C-reactive protein; Bold font indicates statistically significant correlation (*p* < 0.05).

## Data Availability

The original contributions presented in this study are included in the article. Further inquiries can be directed to the corresponding author.

## Abbreviations

The following abbreviations are used in this manuscript:

AITD	Autoimmune Thyroid Diseases
AA	Arachidonic Acid
ATG	Anti–thyroglobulin Antibodies
ATPO	Anti–thyroid Peroxidase Antibodies
